# Metabolic response of tobacco leaves to dry-bulb temperature variation during flue curing: a nontargeted metabolomic approach

**DOI:** 10.3389/fpls.2026.1767521

**Published:** 2026-04-16

**Authors:** Shengjiang Wu, Liping Chen, Jie Zhang, Kesu Wei, Bin Wei, Yunxia Li, Wenxuan Quan, Chaochan Li

**Affiliations:** 1 Guizhou Academy of Tobacco Science, Upland Flue-Cured Tobacco Quality & Ecology Key Laboratory of China Tobacco, Guiyang, Guizhou, China; 2 Guizhou Branch Company of China Tobacco Corporation, Guiyang, China; 3 Guizhou Provincial Key Laboratory for Information Systems of Mountainous Areas and Protection of Ecological Environment, Guizhou Normal University, Guiyang, China

**Keywords:** dry-bulb temperature, flue-curing, lipid metabolism, metabolic pathways, UPLC-MS/MS

## Abstract

This study evaluated the impact of two dry-bulb temperatures—a lower temperature of 44 °C (LT) and a higher temperature of 46 °C (HT)—applied during the color-fixation stage of flue-curing on the metabolomic reprogramming of tobacco (*Nicotiana tabacum* L.) leaves. Fresh leaves were used as controls and compared with leaves cured under the two temperature regimes using a nontargeted metabolomics approach.

**Results:**

A total of 7,233 differentially expressed metabolites (DEMs) were identified across treatments. Compared with fresh leaves, 3,017 and 3,032 DEMs were detected in the 44 °C and 46 °C groups, respectively, while 1,184 metabolites differed between the two curing temperatures (HT vs LT). Pathway enrichment analysis indicated that lipid metabolism, fatty acid biosynthesis, and hormone signal transduction were significantly affected during curing. Notably, malonic acid, indole-3-acetic acid, and docosahexaenoic acid showed temperature-responsive variation, suggesting coordinated regulation of carbon allocation, lipid remodeling, and senescence-related signaling. The higher temperature treatment (46 °C) induced distinct metabolic shifts, including enhanced accumulation of specific lipid- and organic acid-related metabolites, which were associated with increased leaf stiffness after curing. In contrast, curing at 44 °C promoted more balanced metabolic adjustment and moderated lipid oxidative processes.

**Conclusions:**

These findings provide metabolic evidence that subtle differences in color-fixation temperature can significantly influence biochemical coordination and structural traits in tobacco leaves, offering mechanistic insight for optimizing curing temperature to improve leaf quality.

## Graphical Abstract

**Figure:**
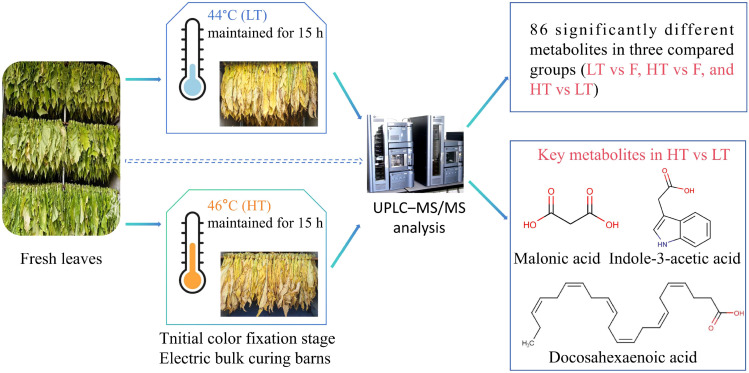

## 1 Introduction

Tobacco (*Nicotiana tabacum* L.) is an economically important crop and a widely used model for studies on plant genetics, breeding, and secondary metabolism (Kinay et al., 2020; Wu et al., 2021). Leaf curing is a decisive postharvest process that determines final industrial quality. It comprises three sequential stages—yellowing, color-fixation (flue-curing), and stem drying—during which extensive biochemical reprogramming occurs (Hu et al., 2021). Carbohydrates, proteins, lipids, and other macromolecules undergo coordinated degradation and transformation, generating metabolites essential for aroma formation, color development, and sensory quality (Chen et al., 2019; Banožić et al., 2020). However, curing efficiency is highly sensitive to environmental parameters, particularly temperature, duration, and humidity, as well as intrinsic differences in raw leaf traits (Liu et al., 2022c; Zong et al., 2022; Jia et al., 2023).

Among these factors, temperature regulation during the color-fixation stage is especially critical, as it governs water loss rate, enzymatic activity, and metabolic flux redistribution. Dry bulb temperatures between 44 °C and 48 °C are considered optimal for effective color fixation and aroma precursor development (Abubakar et al., 2003; Hu et al., 2021). Within this range, lipid remodeling and oxidative metabolism are actively induced, contributing to volatile compound formation. Fatty acid metabolism, in particular, plays a central role in curing-induced aroma generation. Oxidation of polyunsaturated fatty acids via the lipoxygenase pathway produces C6 aldehydes and related volatiles that define characteristic tobacco aroma notes (Wei et al., 2024). Variations in metabolites such as docosahexaenoic acid (DHA) may therefore reflect intensified lipid remodeling processes. In parallel, malonic acid, a key precursor in fatty acid biosynthesis, regulates carbon allocation toward lipid metabolism and may indirectly influence aroma precursor production and sugar–acid balance (Sun et al., 2023). Furthermore, indole-3-acetic acid (IAA) participates in senescence regulation, chlorophyll degradation, and metabolic reprogramming during curing; its dynamic changes are closely associated with yellowing progression and cellular structural modification (Chen et al., 2025). Collectively, lipid-derived metabolites, carbon metabolic intermediates, and hormonal regulators form an interconnected metabolic network that mediates tobacco quality formation under different temperature regimes.

Improper temperature control during color fixation—such as excessive heating rates or unstable thermal maintenance—can disrupt this metabolic coordination (Zou et al., 2019). Brief stabilization periods and rapid temperature increases may lead to incomplete macromolecule degradation, reduced aroma formation, and undesirable physical traits, including leaf stiffness, poor softness, variegation, and uneven surface morphology (Condorí et al., 2020; Meng et al., 2024). Previous studies have demonstrated that excessively high dry bulb temperatures impair volatile accumulation and sensory quality (Cao et al., 2017). When maintained within 44 °C–48 °C under appropriate humidity, enzymatic activity remains active, water loss remains moderate (<50%), and chemical components are more harmonized, resulting in improved flue-cured tobacco quality (Chen et al., 2021; Meng et al., 2024).

Therefore, this study aimed to elucidate the metabolic responses of tobacco leaves to key color-fixation temperatures (44°C and 46°C). By integrating metabolite profiling with quality trait evaluation, we sought to clarify the biochemical pathways and molecular mechanisms underlying temperature-induced differences in leaf stiffness and quality formation. Ultimately, identifying an optimized dry bulb temperature is expected to reduce the proportion of stiff leaves and improve the stability and quality of the flue-curing process.

## 2 Materials and methods

### 2.1 Experimental site and materials

We selected upper leaves from the 2nd to the 3rd position from the top of tobacco plants as our experimental materials from 2022 to 2023. All selected leaves, harvested during the mature period, shared a consistent phenotype in terms of maturity, color, and size. The experimental curing barn, designed by the Guizhou Institute of Tobacco Science, consisted of 6 dense electric bulk curing barns measuring 3.5 m in length and 1.35 m in width. Three barns served as the lower temperature (LT, 44 °C), and three served as the higher temperature (HT, 46 °C), each with six replicates. Tobacco samples were placed on the second layer from the top of the curing barn. Four rods of tobacco leaves were loaded through the tobacco loading door, with one rod placed in each of the left and right paths.

### 2.2 Experimental design

A three-stage curing process was implemented using two different batches of curing barns. During the initial color-fixing stage, one batches of curing barns was set at 44 °C (LT) and maintained for 15 h, while the other was set at 46 °C (HT) and maintained for 15 h. The remaining curing stages followed standard protocols as described by Chen et al. (2021) and Sun et al. (2023). Fresh leaves (F) were therefore subjected to two different dry bulb temperatures (44 °C and 46 °C) during the initial color-fixing period to evaluate temperature-dependent effects on curing performance. Samples were collected from fresh leaves (F) and at the end of the flue-curing process under the 44 °C (LT) and 46 °C (HT) treatments, with six replicates (n = 6) for each condition.

### 2.3 UPLC–MS/MS analysis

UPLC–MS analysis: This experiment used a Waters 2777C UPLC (Waters, USA) series Q Exactive HF high-definition mass spectrometer (USA) to separate and detect metabolites. This test condition is the same as the experimental conditions and detection instruments established by the BGI HR-PMDB database (Bgi Genomics Co., Ltd.).

For metabolic samples preparation, 50 μg sample was added to a 1.5 ml Eppendorf tube, 800 μL of precooled extract solution (methanol:H_2_O = 7:3, V/V) and 20 μL of internal standard 1 (IS 1) were added. A weaving grinding machine was used for 10 minutes at 50 Hz, after which the samples were subjected to ultrasonication in a water bath for 30 minutes. After 1 hour at -20 °C, the extract was centrifuged for 15 minutes at a speed of 14,000 RPM at 4 °C. A 0.22 μm membrane was used to filter 600 μl of the liquid, and the quality control (QC) samples were mixed with 20 μL of filtering solution from each sample to evaluate the repetitiveness and stability of the analysis. The filtered samples and mixed QC samples were transferred to a 1.5 mL sample bottle for instrument operation. When the instrument was analyzed, a QC sample was inserted after every ten experimental samples to monitor the repetitiveness of the experimental samples via the same processing method.

The UPLC–MS–MS (Waters Co., Milford, MA, USA) setup utilized a Hypersil GOLD aQ column (1.9 μm, 2.1 × 100 mm). The mobile phase comprised two components: an aqueous solution containing 0.1% formic acid (liquid A) and acetonitrile containing 0.1% formic acid (liquid B). The elution gradient was programmed as follows: 0–2 min, 5% Liquid B; 2–22 min, 5% to 95% B; 22–27 min, 95% B; 27–27.1 min, 95% to 5% B; and 27.1–30 min, 5% B. The flow rate was 0.3 mL/min, with a column temperature of 40 °C and an injection volume of 5 μL.

Q Exactive mass spectra were scanned within the mass–charge ratio ranges of 125–1500 for positive ions and 100–1500 for negative ions, yielding a primary resolution of 120000. AGC targets were set to 1e^6^ for positive ions and 3e^6^ for negative ions, with a maximum injection time of 100 ms. Fragmentation data (Top3 method) were collected using a secondary resolution of 30000, AGC targets of 2e^5^ for positive ions and 1e^5^ for negative ions, a maximum injection time of 50 ms, and stepped collision energies of 20, 40, and 60 eV. The electrospray ionization (ESI) parameters were set according to the method outlined by Wu et al. (2024).

### 2.4 Metabolite analysis

The raw mass spectrometry data were processed with Compound Discoverer 3.3 software (Thermo Fisher Scientific, Waltham, MA, USA) for initial spectrum analysis. The data were subsequently imported into Metax for preprocessing and in-depth analysis. The preprocessing steps included normalization of the data via probability quotient normalization (PQN) to calculate relative peak areas (Di Guida et al., 2016); batch effect correction via quality control-based robust LOESS signal correction (QC-RLSC); and exclusion of compounds with a coefficient of variation (CV) exceeding 30% across all the quality control samples (Dunn et al., 2011). For semiquantitative metabolite analysis, the mass spectrometry data were annotated against the BGI Metabolome DataBase (BMDB), MzCloud, and ChemSpider online databases (**Supplementary Table 1**). Principal component analysis (PCA) and orthogonal partial least squares-discriminant analysis (OPLS-DA) were utilized for subsequent data analysis.

The variable importance in the projection (VIP) is based on the OPLS-DA model. Moreover, the P value/FDR or FC value that can be analyzed by a single variable analysis can be further selected to screen for differences. The different metabolite screening criterion used was a VIP ≥ 1 in the OPLS-DA model. Fold change ≥ 1.2 or ≤0.83, q value < 0.05 was used as a measure of statistical significance in differential metabolites between samples.

### 2.5 KEGG annotation and enrichment analysis of differential metabolites

The identified metabolites were annotated via the KEGG compound database (http://www.kegg.jp/kegg/compound/), and the annotated metabolites were subsequently mapped to the KEGG pathway database (http://www.kegg.jp/kegg/pathway.html). The functional injection and release pathways were determined via the KEGG pathway database, and the main biochemical metabolic pathways and signal transduction pathways associated with the metabolites were determined.

### 2.6 Statistical analyses

MetaX software was used for principal component analysis and partial least squares discriminant analysis of the metabolite data. Adobe Illustrator (v26.4.1) was used to integrate the graphics.

## 3 Results

### 3.1 PCA of total metabolites in tobacco leaves

**Figure 1:**
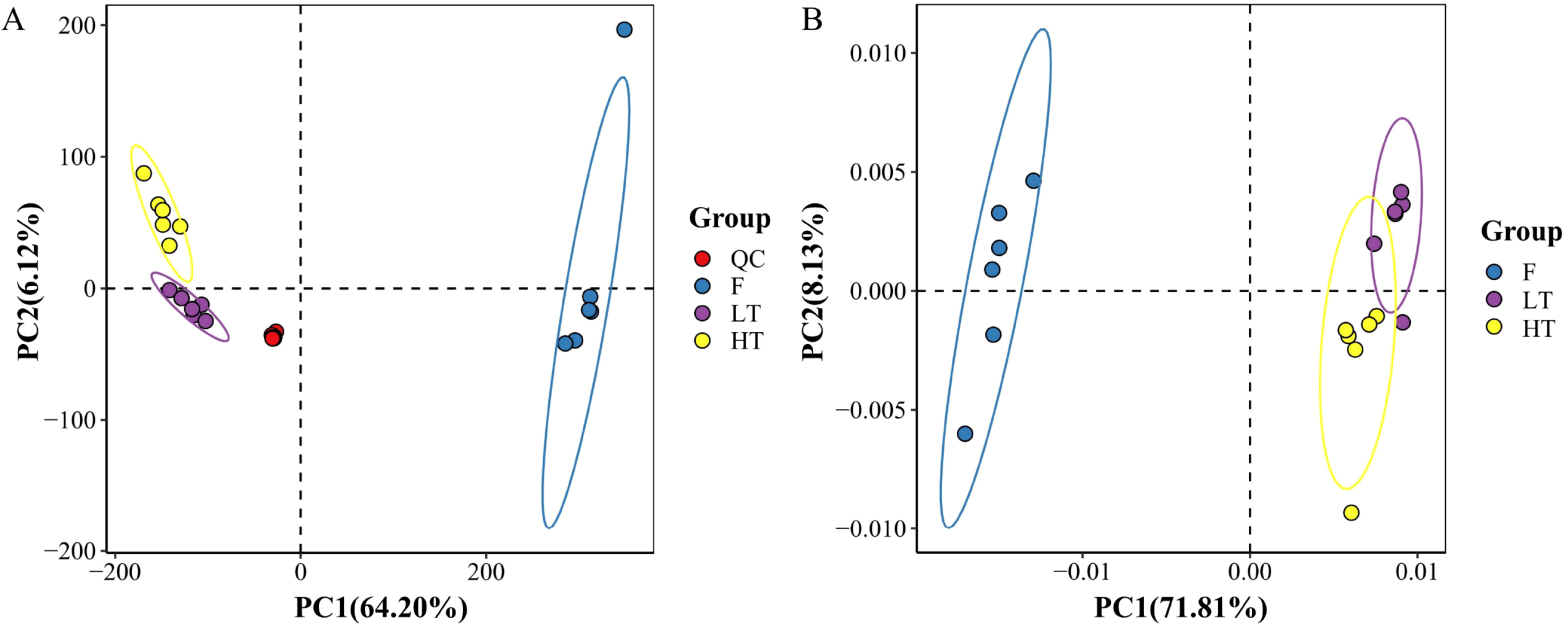
PCA scores of mass spectral data for each group of samples. **(A)** PCA scores with QC samples, **(B)** PCA scores without QC samples. The ellipse in the PCA score chart is the 95% confidence interval. Each point represents a sample, different groups are marked with different colors, and the ellipse is a 95% confidence interval.

A PCA model was constructed to assess metabolomic variation across sample groups, including QC samples (n=9). The distinct clustering of the QC, F, LT, and HT groups along PC1 explained 64.20% of the total variance, indicating that significant metabolite profile differences were induced by the curing temperature. Tightly aggregated QC samples confirmed minimal technical variability and high experimental reproducibility (**Figure 1A**).

From the PCA results without QC samples, the samples of different groups (F, LT, HT) exhibit distinct clustering patterns in the PCA score plot, indicating that these groups of samples have significant differences in relevant measured indicators, and the samples within each group have a certain degree of homogeneity, with good discrimination between groups. The first principal component (PC1) explains 71.81% of the variance, and the second principal component (PC2) explains 8.13% of the variance (**Figure 1B**). These two principal components together account for a large proportion of the variance, effectively reflecting the overall differences among the samples.

### 3.2 Identification, classification, and metabolic pathways of metabolites

**Figure 2:**
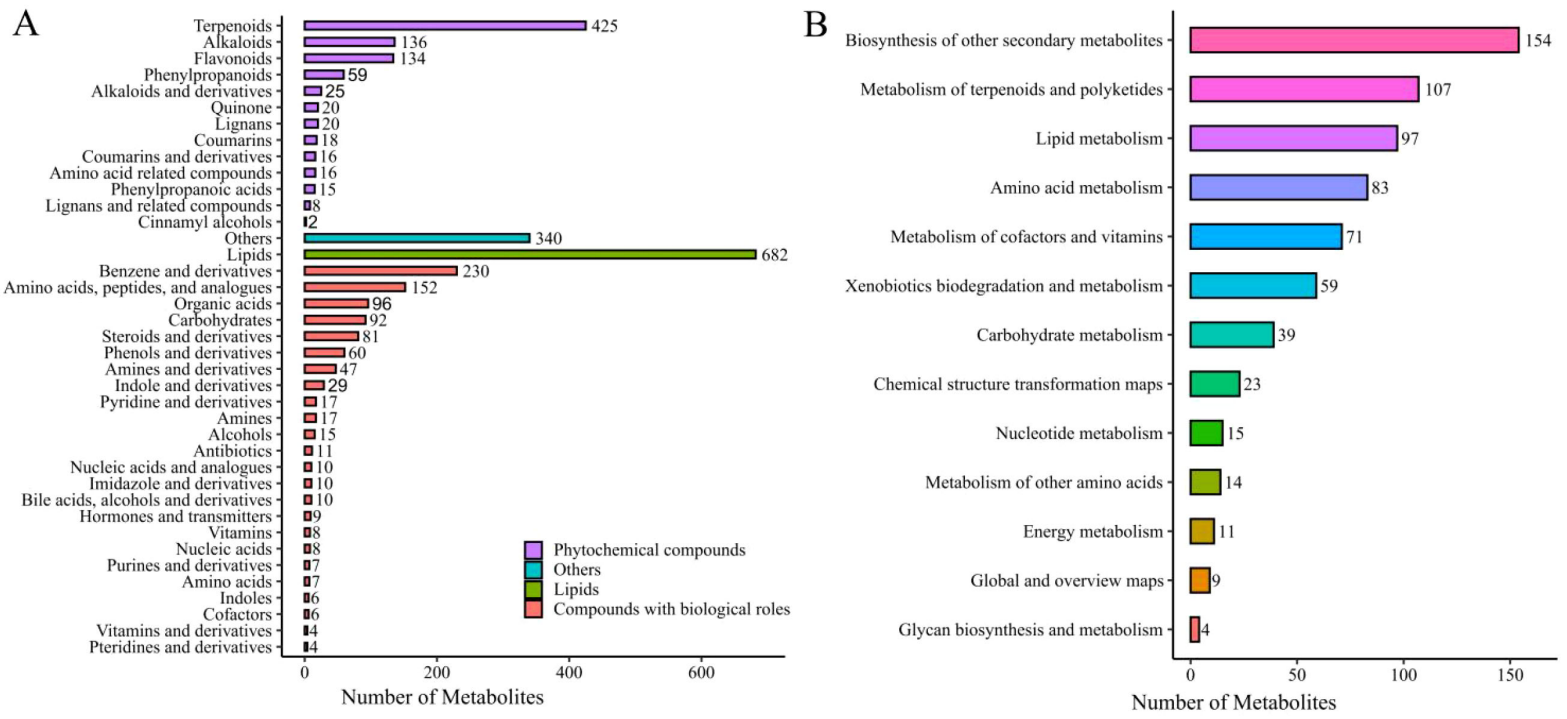
Metabolite classification diagram **(A)**, and metabolic pathway classification diagram **(B)**.

A total of 7042 metabolites were successfully matched and classified into 39 categories (**Figure 2A**). Lipids were the most abundant metabolites, followed by coumarin and its derivatives, terpenoids, others, benzene and derivatives, amino acids, peptides, and analogs, alkaloids, flavonoids, organic acids, carbohydrates, steroids and derivatives, phenols and their derivatives, phenylpropanoids, amines and their derivatives, and indoles and their derivatives.

This study annotated 686 metabolites in KEGG pathways, which were distributed across 13 metabolic pathways (**Figure 2B**). The biosynthesis of other secondary metabolites pathway contained the highest number of annotated metabolites, followed by terpenoid and polyketide metabolism, lipid metabolism, amino acid metabolism, and metabolism of cofactors and vitamins. These metabolic pathways provide insights into how specific metabolites influence the quality characteristics of cured tobacco leaves.

### 3.3 Differentially abundant metabolite analysis among different compared groups

#### 3.3.1 Differentially abundant metabolite screening

**Figure 3:**
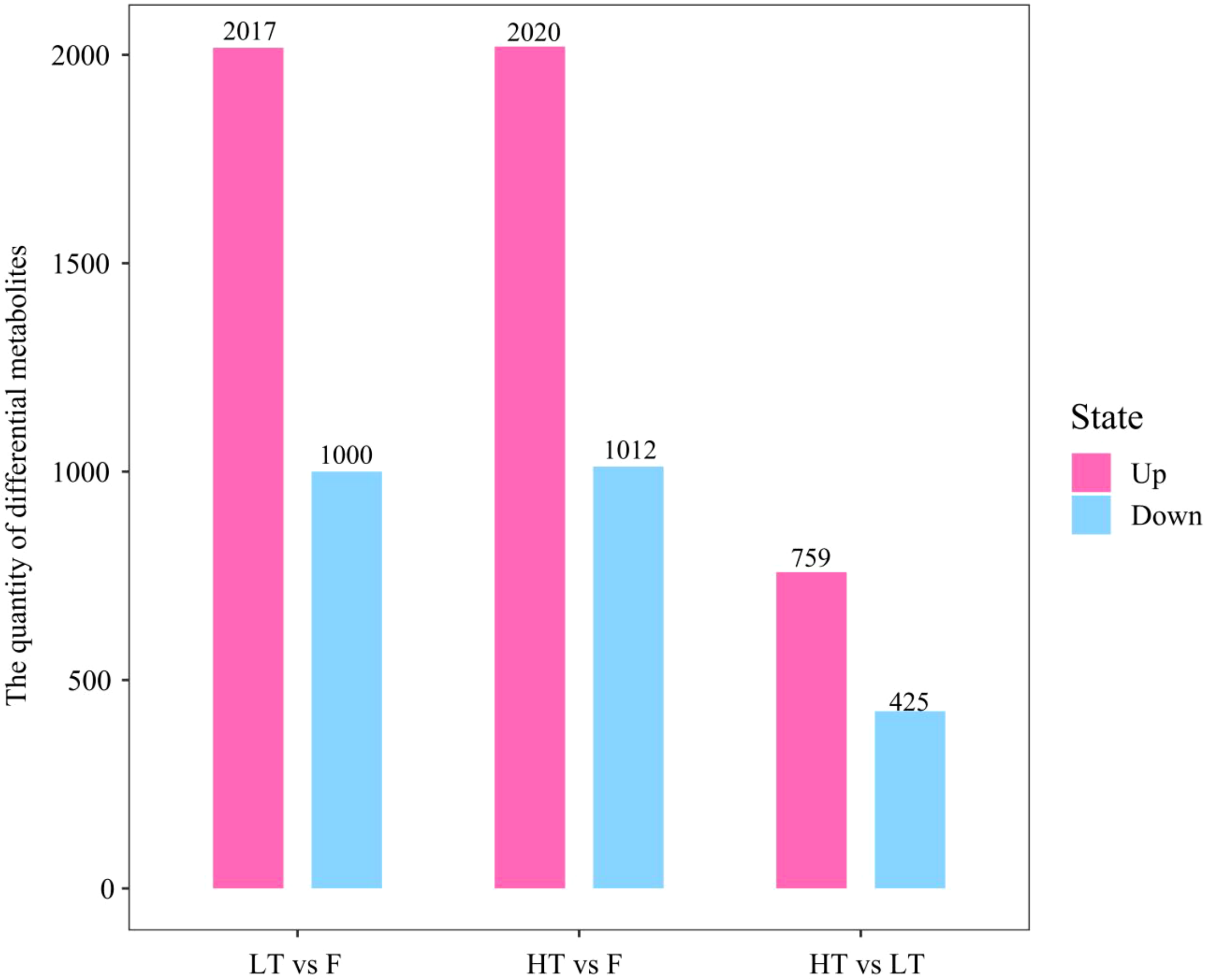
Statistical chart of the quantity of differential metabolites. Up: number of upregulated differential metabolites, Down: number of downregulated differential metabolites.

A total of 7233 differentiated metabolites were identified across three compared groups: 3017 differentially expressed metabolites (DEMs) in LT vs F (2017 upregulated, 1000 downregulated), 3032 DEMs in HT vs F (2020 upregulated, 1012 downregulated), and 1184 DEMs in HT vs LT (759 upregulated, 425 downregulated) (**Figure 3**).

**Figure 4:**
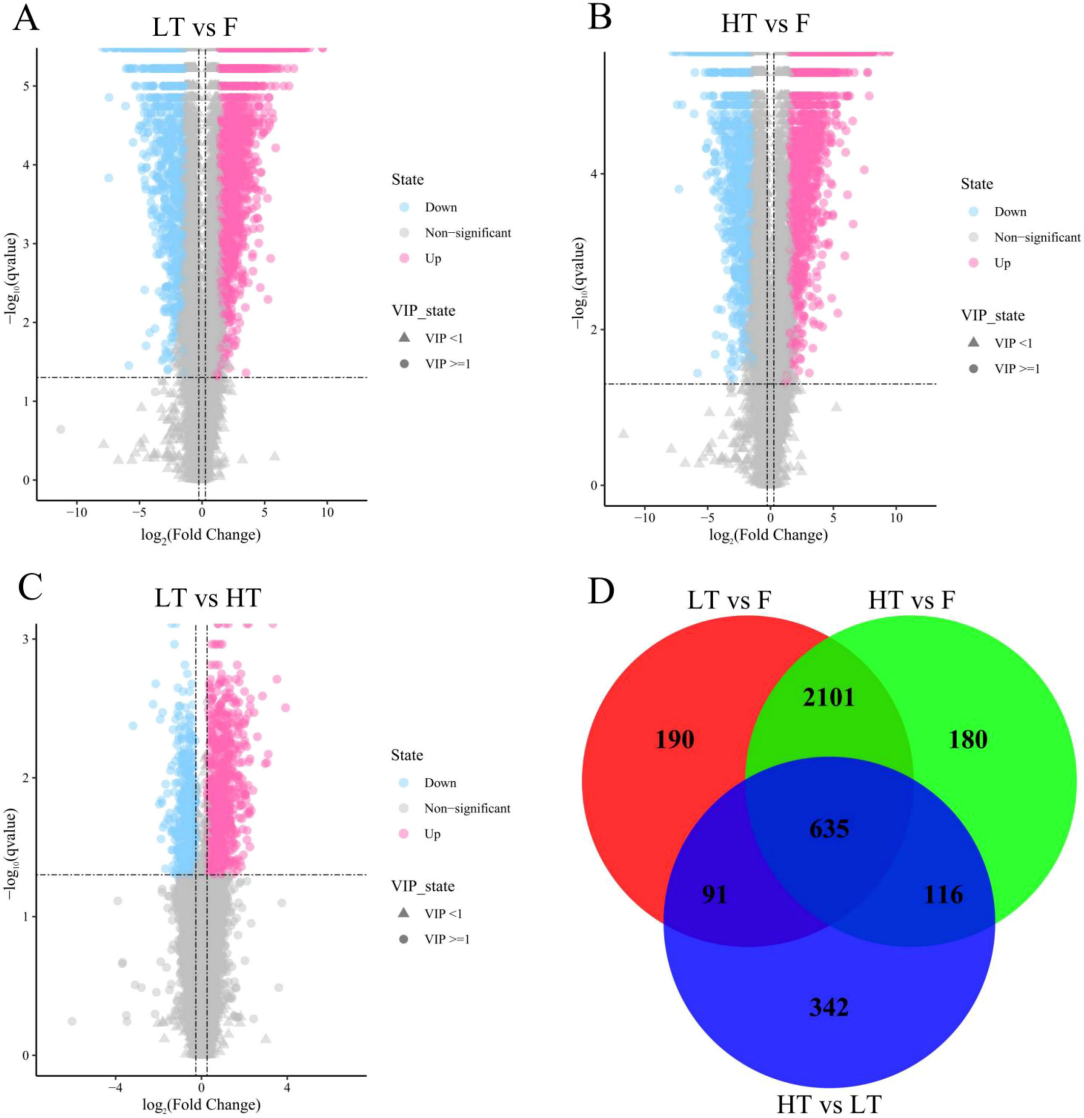
Volcanic diagram **(A–C)** and Venn diagram statistics **(D)** of differentially abundant metabolites of tobacco samples in different compared groups. Blue represents the significantly downregulated metabolite, red represents the significantly upregulated metabolite, the circle represents the metabolite with a VIP greater than or equal to 1, the triangle represents the metabolite with a VIP less than 1, and the insignificant metabolite is gray.

The number of compounds detected in LT vs F, HT vs F was not significantly different, with 3017 and 3032 DEMs, respectively. However, leaves cured at HT presented a greater number of upregulated metabolites than did those cured at LT. Notably, the number of differentially abundant metabolites in tobacco leaves cured at HT vs LT was significantly lower than that in LT vs F, HT vs F (**Figures 4A–C**). Further analysis via a Venn diagram revealed that 635 differentially abundant metabolites were shared among the three compared groups (**Figure 4D**). This overlap suggests that these metabolites respond positively to complex adjustments in biochemical processes and metabolic pathways during tobacco curing.

#### 3.3.2 KEGG pathway annotation and enrichment analysis of differential metabolites

On the basis of the KEGG annotations, the differential metabolites identified in each compared group were allocated to various metabolic pathways, providing insights into the biochemical processes influencing tobacco leaf quality during the curing process.

**Figure 5:**
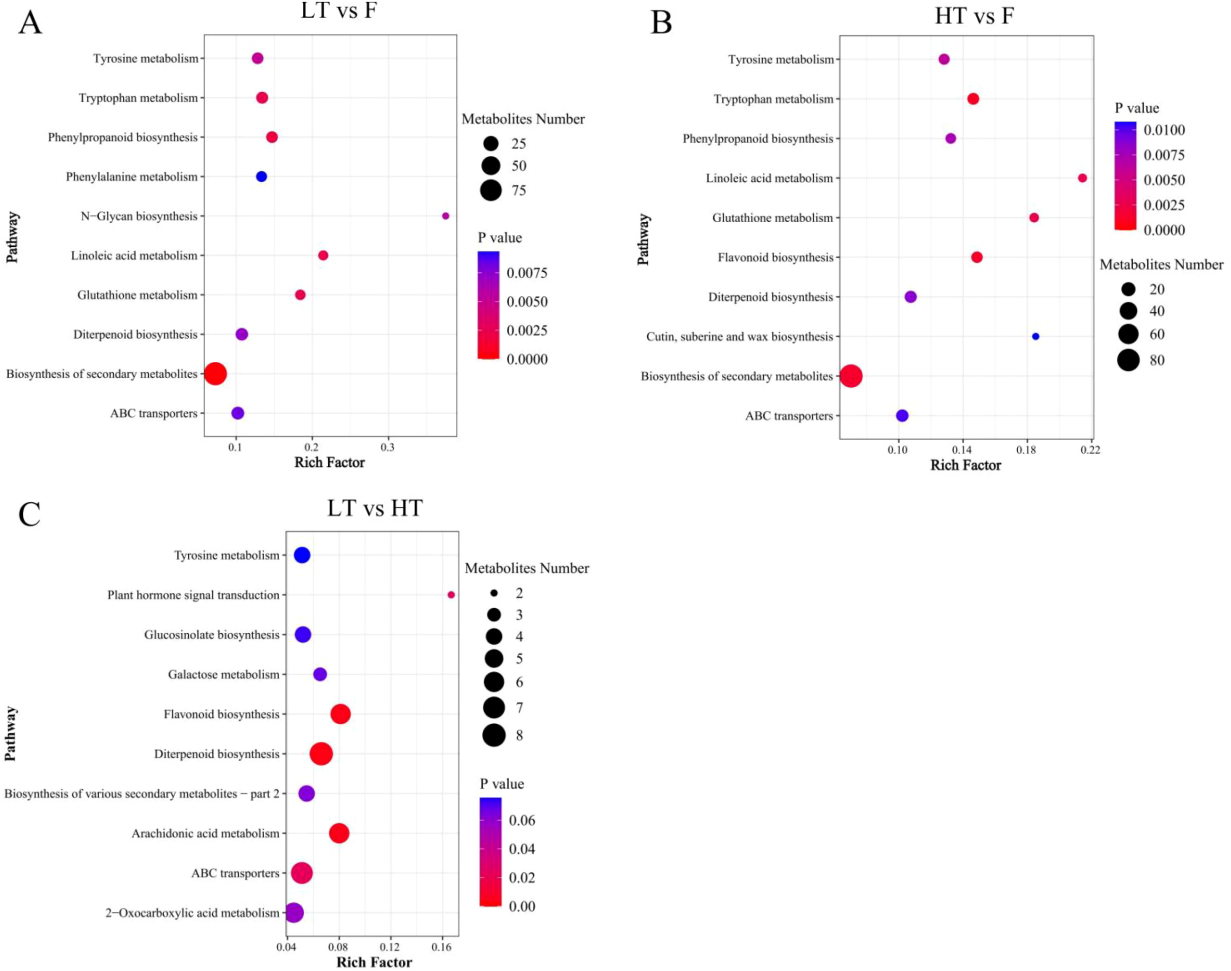
Bubble plot for pathway enrichment analysis of differential metabolites in different compared groups. **(A)** LT vs F, **(B)** HT vs F, **(C)** HT vs LT. The X-axis enrichment RichFactor is the number of different metabolites annotated to the pathway divided by the number of all metabolites of the pathway. The greater this value is, the greater the proportion of different metabolites annotated to the pathway. The bubble size represents the number of differentially abundant metabolites annotated to the pathway. The darker colors represent higher enrichment levels.

In the LT vs F group, 172 differentially abundant metabolites were categorized into 10 metabolic pathways. The most prominent pathways included secondary metabolite biosynthesis, phenylpropanoid biosynthesis, linoleic acid metabolism, tryptophan metabolism, and glutathione metabolism, with secondary metabolite biosynthesis being the most enriched (**Figure 5A**). Similarly, in the HT vs F group, 174 differentially abundant metabolites were allocated across 10 metabolic pathways. The key pathways identified were tryptophan metabolism, flavonoid biosynthesis, secondary metabolite biosynthesis, linoleic acid metabolism, and glutathione metabolism, with tryptophan metabolism being the most significant (**Figure 5B**). In the HT vs LT compared group, 50 differentially abundant metabolites were distributed among 10 metabolic pathways. Diterpenoid biosynthesis emerged as the most significant pathway, followed by flavonoid biosynthesis, arachidonic acid metabolism, ABC transporters, and plant hormone signal transduction, indicating its potential importance in the physiological and biochemical responses of flue-cured tobacco leaves when cured at 44 °C compared with those when cured at 46 °C (**Figure 5C**).

#### 3.3.3 Metabolite analysis of significant different metabolites

**Figure 6:**
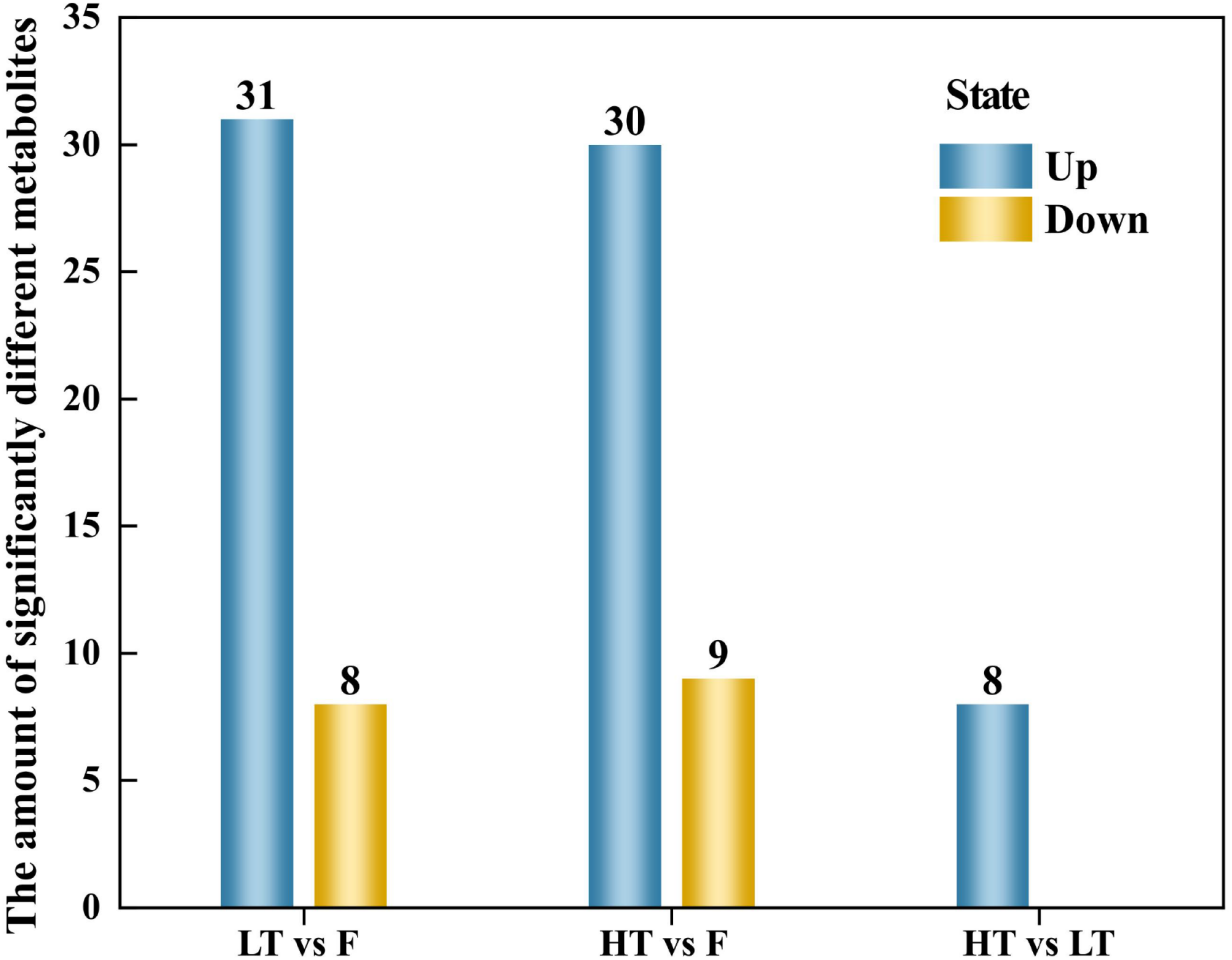
Significantly different metabolites in the positive and negative ion modes.

Across all the compared groups, 86 significantly different metabolites were identified, which were primarily categorized into lipids, aromatic compounds, organic acids and their derivatives, phenols, nucleosides, heterocyclic compounds, terpenes, alkaloids, and organic oxides. Significant changes in metabolite profiles were observed during tobacco curing at different temperatures. Among the 29 significantly different lipid metabolites, 25 were upregulated across all the compared groups (LT vs F, HT vs F, HT vs LT), including desoxycortone, taurochenodeoxycholic acid, and docosapentaenoic acid. However, lithocholic acid was downregulated in HT vs F, whereas docosahexaenoic acid was upregulated in HT vs LT (**Figure 6**; **Supplementary Table 2**).

Among the nine significantly different aromatic metabolites, seven were upregulated in both the LT vs F groups and the HT vs F groups. Methadone exhibited complex regulation, being upregulated in LT vs F and HT vs F but downregulated in HT vs LT. Other aromatic compounds, such as 2-amino-3-methoxybenzoic acid and 2,3-dihydroxybenzoic acid, were upregulated in LT vs F. Among the organic acids and their derivatives, 8 metabolites in this category showed significant differential expression. Among them, the metabolites malonic acid, indole-3-acetic acid and docosahexaenoic acid were significantly enriched in the three compared groups. Specifically, malonic acid and indole-3-acetic acid were increased in the LT vs F and HT vs F groups. Conversely, indole-3-acetic acid was downregulated in the LT vs F and HT vs F groups but upregulated in the HT vs LT group. These three metabolites may be the key regulatory metabolites that regulate physiological and biochemical reactions in the tobacco curing process.

Additionally, eight metabolites were significantly upregulated in the HT vs LT group. It consists of docosapentaenoic acid, docosahexaenoic acid, calcitriol, 7-methyl xanthine, malonic acid, n-acetyl-DL-glutamic acid, 1,4-androstenedione-3,17-dione, and indole-3-acetic acid (**Supplementary Table 2**). The preliminary screening results revealed that these substances may be the key components of stiff tobacco leaves and that their contents are significantly enriched in the dry-bulb high-temperature (46 °C) process during the curing and color fixing periods.

## 4 Discussion

Tobacco metabolites undergo significant changes before and after curing, necessitating detailed research to increase tobacco quality and promote sustainable tobacco industry practices. Before curing, fresh tobacco primarily contains sugars, amino acids, and organic acids, which are vital for growth, development, aroma, and taste (Zhang et al., 2013; Liu et al., 2022a). These metabolites undergo complex chemical reactions during curing, such as the Maillard reaction and esterification (Zhao et al., 2014; Meng et al., 2022). These changes not only affect tobacco quality and taste but also impact its physiological functions and health effects.

Research has shown that post curing, tobacco leaf metabolites exhibit increased aroma components and pigment formation. Aroma components result mainly from the pyrolysis and oxidation of sugars, amino acids, and other compounds during curing, producing esters, aldehydes, ketones, and other aromatic compounds that contribute to the distinctive aroma and flavor of tobacco (Ma et al., 2019; Zou et al., 2023). Additionally, pigment substances form from the degradation and transformation of carotenoids, flavonoids, and other compounds during flue-curing. These metabolites undergo oxidation, reduction, and other reactions to produce various pigments, such as brown pigments and melanin, influencing tobacco color and appearance (Wu et al., 2020; Hu et al., 2021; Liu et al., 2022b). In the present investigation, we identified a comprehensive set of 7233 differentially abundant metabolites in tobacco samples. Comparative analyses between the LT vs F, HT vs F, and HT vs LT groups revealed 3017, 3032, and 1184 DEMs, respectively. The majority of these metabolites were significantly upregulated across the different compared groups. Functional annotation through KEGG metabolic pathway enrichment indicated that the differential metabolites produced during the curing process were predominantly involved in four metabolic pathways: secondary metabolite biosynthesis, tryptophan metabolism, linoleic acid metabolism, and glutathione metabolism.

A total of 86 metabolites were significantly differentially expressed across the LT vs F, HT vs F, and HT vs LT groups. These metabolites are primarily classified into lipids, aromatic compounds, organic acids and their derivatives, phenolics, nucleosides, heterocyclic compounds, terpenoids, alkaloids, and organic oxides. Metabolomic studies have highlighted lipid metabolism and hormone signaling as critical regulators of tobacco leaf quality formation during flue-curing. Differential metabolite profiling during the color-fixation stage (44°C vs 46°C) identified malonic acid, indole-3-acetic acid (IAA), and docosahexaenoic acid (DHA) as significantly enriched compounds associated with physiological adjustment and structural remodeling of tobacco leaves (Sun et al., 2023; Wei et al., 2024; Chen et al., 2025). During flue-curing, tobacco leaves undergo stresses such as high temperature and drought, leading to the accumulation of cellular reactive oxygen species (ROS). Excessive ROS can damage cell membranes and affect lipid metabolism, potentially increasing the oil and aroma contents in flue-cured tobacco leaves, thereby increasing smoking quality (Mittler, 2002; Dunkle et al., 2016). This study found that baking at lower temperatures during the curing and fixing stage is more conducive to improving the quality of flue-cured tobacco, reducing its hardness and the accumulation of related metabolites. It should be noted that the present study is based on metabolomic association analysis, although malonic acid, IAA, and DHA showed temperature-responsive variation correlated with leaf stiffness, direct causal relationships cannot be concluded from metabolite abundance alone. Future studies involving exogenous metabolite application under controlled curing conditions, combined with enzyme activity assays and cell wall structural analyses, will be necessary to establish a mechanistic link between metabolite dynamics and leaf mechanical properties. The absence of intermediate sampling during the curing process limits our ability to resolve temporal response patterns of key metabolites. Therefore, whether the observed differences in malonic acid, IAA, and DHA represent rapid early responses to temperature variation or cumulative metabolic adjustments cannot be conclusively determined. Future time-course studies incorporating multiple sampling points during the color-fixation stage will be necessary to clarify the dynamic regulation and temperature sensitivity of these metabolites.

This study focused on subtle differences in dry-bulb temperature (44 °C vs. 46 °C) during the color-fixation stage and systematically characterized the associated metabolic responses using a nontargeted metabolomics approach. The results demonstrate that even a 2 °C variation can induce distinct metabolic reprogramming, particularly in lipid metabolism–related pathways. Differential metabolites, including malonic acid, indole-3-acetic acid, and docosahexaenoic acid, were identified as temperature-responsive components potentially linked to carbon allocation, hormonal regulation, and membrane lipid remodeling. These coordinated metabolic adjustments may underlie the observed differences in leaf stiffness and overall quality. Collectively, our findings provide metabolic evidence that precise temperature control during the color-fixation stage is critical for maintaining biochemical balance and optimizing tobacco leaf texture and quality.

## 5 Conclusions

This study revealed that lowering the dry bulb temperature led to timely softening of the leaf texture and reduced the proportion of stiffness in the cured leaves. This modification improved the metabolite characteristics of the tobacco leaves. In the comparative analysis of three groups (LT vs F, HT vs F, and HT vs LT), a total of 7233 metabolites exhibited differential abundance, 86 of which were significantly altered, predominantly within the lipid metabolism pathways. Notably, eight of these metabolites, identified in the HT vs LT group, are potential key contributors to the physiological response associated with leaf stiffness. Malonic acid, indole-3-acetic acid, and docosahexaenoic acid have emerged as potential key metabolites of the physiological and biochemical responses of tobacco leaves before and after curing at 44°C and 46°C, warranting further investigation to confirm their regulatory roles. These findings offer further insights into the physiological and biochemical changes, as well as the metabolic activities, associated with tobacco leaf curing.

## Data Availability

The original contributions presented in the study are included in the article/**Supplementary Material**. Further inquiries can be directed to the corresponding author.

## Supplementary material

Supplementary Table 1 Metabolite identification and relative abundance information for all samples.

Supplementary Table 2 Significantly different metabolites screened in three compared groups.

## Glossary

UPLC-MS: ultra performance liquid chromatography-tandem mass spectrometry
LC–MS: liquid chromatography–mass spectrometry
DEMs: differentially expressed metabolites
KEGG: Kyoto encyclopedia of genes and genomes
CV: coefficient of variation
LT: low temperature
HT: high low temperature
F: fresh leaf.
